# Childhood BMI is inversely associated with pubertal timing in normal-weight but not overweight boys

**DOI:** 10.1093/ajcn/nqy201

**Published:** 2018-10-13

**Authors:** Maria Bygdell, Jenny M Kindblom, Jimmy Celind, Maria Nethander, Claes Ohlsson

**Affiliations:** 1 Centre for Bone and Arthritis Research, Department of Internal Medicine and Clinical Nutrition, Institute of Medicine; 2 Bioinformatics Core Facility, Sahlgrenska Academy at University of Gothenburg, Gothenburg, Sweden

**Keywords:** childhood overweight, childhood BMI, pubertal timing, age at peak height velocity, cohort study

## Abstract

**Background:**

An inverse association between childhood body mass index (BMI; in kg/m^2^) and pubertal timing is well established for girls. Among boys, studies are scarce and the results inconclusive.

**Objective:**

We aimed to determine the association between childhood BMI and age at peak height velocity (PHV) in boys.

**Design:**

We collected height and weight measurements between 6.5 and 22 y of age for boys born 1945–1961 (original cohort; *n* = 31,971; mean ± SD childhood BMI: 15.74 ± 1.41; age at PHV: 14.06 ± 1.11 y) and 1981–1996 (replication cohort; *n* = 1465; childhood BMI: 16.47 ± 2.06; age at PHV: 13.71 ± 1.08 y) attending schools in Gothenburg, Sweden, and examined at mandatory military conscription. Age at PHV was obtained from curve-fitting of measured heights with the use of a modified Infancy-Childhood-Puberty model.

**Results:**

In the original cohort, childhood BMI was inversely associated with age at PHV (*P* < 0.001) and a significant quadratic term for childhood BMI (*P* < 0.001) indicated the nonlinearity of this association. Via piecewise linear regression, we identified a threshold for the association at a childhood BMI of 18.42. A significant inverse association was observed below (β: −0.17 y/BMI unit; 95% CI: −0.18, −0.16 y/BMI unit) but not above (β: 0.02 y/BMI unit; 95% CI: −0.03, 0.06 y/BMI unit) this childhood BMI threshold. For every unit increase in childhood BMI, age at PHV was ∼2 mo earlier up to the childhood BMI threshold. Similar results were observed in the replication cohort, demonstrating a significant inverse association below (β: −0.16; 95% CI: −0.21, −0.11) but not above (β: −0.03; 95% CI: −0.11, 0.05) the childhood BMI threshold. The identified threshold was close to the cutoffs for overweight at 8 y of age, and childhood BMI was inversely associated with age at PHV below but not above the overweight cutoffs.

**Conclusions:**

The present findings establish an inverse association between childhood BMI and pubertal timing in normal-weight but not overweight boys.

## INTRODUCTION

The transition from childhood to adulthood through puberty is one of the milestones in human development. Yet, the regulation of the initiation of puberty is still poorly understood. Among girls, an inverse association between childhood BMI (in kg/m^2^) and pubertal timing is well established and a systematic review of longitudinal studies recently confirmed that higher BMI at 7 and 8 y of age was associated with earlier menarche (1). Moreover, a secular trend of earlier pubertal timing among girls has been observed since the mid-19^th^ century (2), and it has been proposed that the increasing prepubertal BMI during this period could at least partly explain the trend of earlier pubertal timing. Other factors contributing to this trend of earlier pubertal timing might include overall better hygienic conditions and nutrition as well as improved health care (3). It has also been proposed that increased use of endocrine-disrupting chemicals might have contributed to the secular trend of earlier puberty (4).

Because there is no easily defined pubertal event in boys, such as menarche, the association between childhood BMI and male pubertal timing is less evaluated. Conflicting results with both positive and negative associations between childhood BMI and male pubertal timing have been reported. A Danish longitudinal study demonstrated an inverse association between childhood BMI at 7 y of age and age at peak height velocity (PHV) among boys born 1930–1969 who had their height measured between 7 and 15 y of age (5, 6). However, the representativeness of the cohort was only 10% and the study was therefore not population-based. Moreover, because the last height measurement was performed at 15 y of age, the study did not include boys with late puberty. In contrast to the Danish study, a small longitudinal study from the United States with information on BMI and pubertal timing in boys born in 1991 showed that boys with a high BMI between 2 and 11.5 y of age were more likely to display a late pubertal timing (7).

For pubertal assessment, self-report of pubertal demarcations (menarche for girls or, e.g., age at voice break for boys) can be used retrospectively, but self-reported voice break in boys is limited by recall bias. Age at PHV, the age at the maximum growth spurt, is an objective assessment of pubertal timing (8).

The aim of the present study was to evaluate the association between childhood BMI and pubertal timing in boys. We used the well-powered population-based BMI Epidemiology Study (BEST), with information on childhood BMI at 8 y of age and age at PHV available, as an objective assessment of pubertal timing. To adequately calculate age at PHV in an unbiased manner, height measurements before, during, and after the pubertal period are required, and the BEST cohort is the first large-scale study with this combined information available for boys. We hypothesize that there is an inverse association between childhood BMI and age at PHV in boys.

## METHODS

The research performed was not a clinical trial and therefore did not require registration. The prespecified primary endpoint of the present study was age at PHV; this was not changed during the course of the study.

### Study population

**FIGURE 1:**
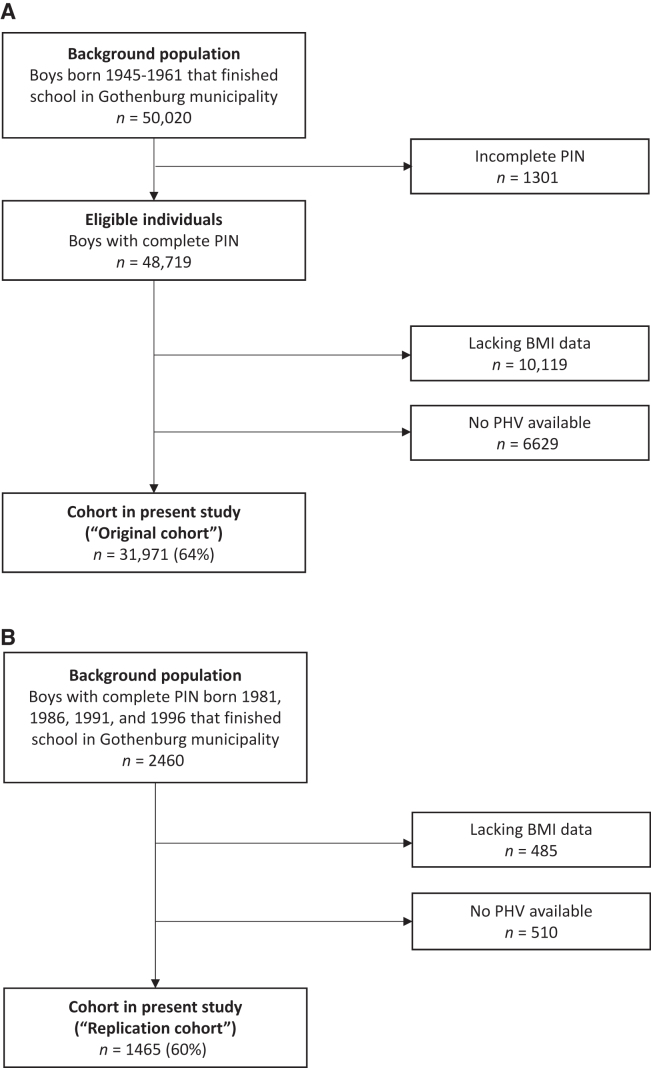
Flow chart of included participants with exclusions in hierarchic order. (A) The original cohort (*n* = 31,971). (B) The replication cohort (*n* = 1465) including boys born consecutively from the start of the year in the included birth years. PHV, peak height velocity; PIN, personal identity number.

The population-based BEST cohort was initiated with the overall aim to determine the role of childhood BMI and pubertal timing for a variety of diseases in adulthood (9–11). The cohort includes individuals that completed school in the Gothenburg municipality and afterwards had their school health record stored at the Archives of City of Gothenburg and Region Västra Götaland. The school health records include data on height and weight from regular health visits at child health care centers and school health care throughout childhood until the children finished secondary school. The health visits include all children in Sweden (>98.5% for school health care from calendar year 1952) (12). For the present study, we collected childhood height and weight from the school health records and height and weight at young adult age from mandatory military conscriptions. Subjects eligible for the present study were those with a school health record in the central archive and a 10-digit personal identity number (PIN). The inclusion criteria for the present study were data being available for calculation of both childhood BMI and age at PHV (**Figure 1**). For the included individuals, we used measurements of height and weight between 6.5 and 22 y of age in boys born 1945–1961 (original cohort; *n* = 31,971; mean ± SD childhood BMI: 15.74 ± 1.41; age at PHV: 14.06 ± 1.11 y) and 1981–1996 (replication cohort; *n* = 1465; childhood BMI: 16.47 ± 2.06; age at PHV: 13.71 ± 1.08 y). All analyses were first performed in the original cohort and then replicated in the replication cohort, which showed higher prevalences of overweight and obesity (**Table 1**).

**TABLE 1:** Childhood BMI and age at PHV in the original cohort and the replication cohort^1^

	*n*	Childhood BMI, kg/m^2^	Age at PHV, y
Original cohort (born 1945–1961)	31,971	15.74 ± 1.41	14.06 ± 1.11
Replication cohort (born 1981–1996)	1465	16.47 ± 2.06	13.71 ± 1.08

^1^Values are means ± SDs unless otherwise indicated. PHV, peak height velocity.

This study was approved by the ethics committee of the University of Gothenburg, Sweden.

### Linkage with register from Statistics Sweden

In Sweden, a PIN is assigned to every citizen at birth or immigration. With the use of the individuals’ PIN, the BEST cohort was linked with the Longitudinal Integration Database for Health Insurance and Labor Market Studies at Statistics Sweden, and country of birth for every study participant and their parents was retrieved.

### Calculations of childhood BMI and age at PHV

Prepubertal childhood BMI was calculated with the use of all paired height and weight measurements in the period between 6.5 and 9.5 y of age and age-adjusted to 8 y of age with the use of a linear regression model.

To adequately calculate age at PHV in an unbiased manner, height measurements before, during, and after the pubertal period are required. We calculated age at PHV according to a modified Infancy-Childhood-Puberty model (13). A good model fit was confirmed through visual inspection of all curves (MB, JMK). Age at PHV was defined as the age at maximum growth velocity during puberty and was estimated by the curve-fitting program.

### Statistical analyses

Associations between childhood BMI and age at PHV were investigated through the use of linear regression. A possible nonlinear association was assessed through the inclusion of a quadratic childhood BMI term in the model. Via a piecewise linear regression model (R-package “segmented” version 3.4.2; http://www.r-project.org) (14) we further explored the association adjusted for birth year and country of birth.

The distribution of childhood BMI was slightly positively skewed and all analyses were therefore repeated with the use of log-transformed childhood BMI, with similar results. To facilitate interpretation, we present all the results generated with the use of the nontransformed childhood BMI.

Analyses that used the piecewise regression were done in R version 3.4.2 (segmented package) (14). For all other statistical analyses, SPSS (IBM SPSS, Armonk) version 24 was used.

## RESULTS

The association between childhood BMI at 8 y of age and age at PHV was first evaluated in a linear regression model. Childhood BMI was inversely associated with age at PHV. Inclusion of a quadratic term for childhood BMI revealed a statistically significant nonlinear association between childhood BMI and age at PHV (*P* < 0.001). The nonlinear association was further explored via a piecewise linear regression under the assumption of one threshold. A threshold for the association at a childhood BMI of 18.42 (95% CI: 17.97, 18.87) was identified.

**FIGURE 2:**
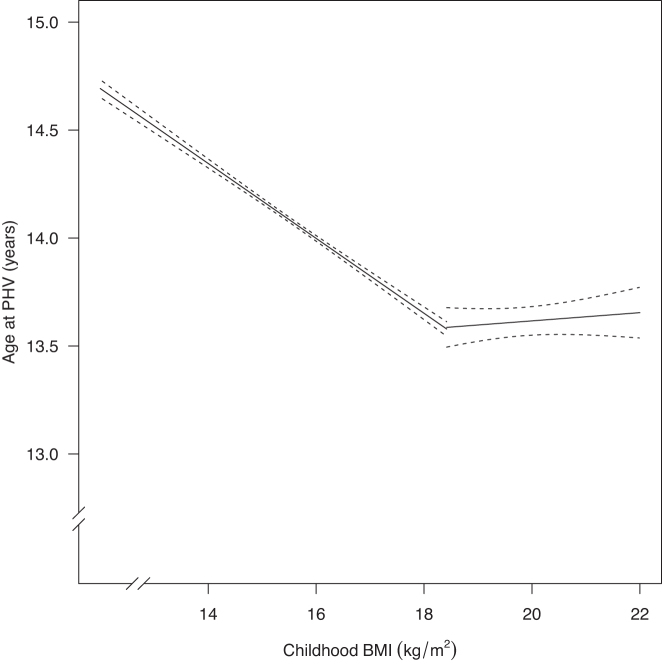
Association between childhood BMI and age at PHV via a piecewise linear regression model, in the original cohort (*n* = 31,971; 95% CIs represented by dotted lines). Via a piecewise linear regression model (R-package “segmented”), we identified a threshold at BMI 18.42 (95% CI: 17.97, 18.87) for the association between childhood BMI and age at PHV, when evaluated in the original cohort adjusting for birth year and country of birth. PHV, peak height velocity.

Below the identified BMI threshold at 18.42, a statistically significant inverse association between childhood BMI and age at PHV was observed (β: −0.17; 95% CI: −0.18, −0.16). In children with a BMI below the threshold at 8 y, age at PHV was 2 mo earlier for every unit increase of childhood BMI. Of note, there was no significant association between BMI and age at PHV among boys with a BMI above the threshold (**Figure 2**; **Table 2**). Similar results were observed in the replication cohort, demonstrating a significant inverse association below (β: −0.16; 95% CI: −0.21, −0.11) but not above (β: −0.03; 95% CI: −0.11, 0.05) the childhood BMI threshold (Table 2). Thus, the association observed in the original cohort was confirmed in a replication cohort including individuals that grew up later and were exposed to the obesity epidemic.

**TABLE 2:** Association between childhood BMI and age at PHV according to the identified childhood BMI threshold estimated from the piecewise linear regression^1^

	Childhood BMI, kg/m^2^	
	<18.42	≥18.42
Original cohort (born 1945–1961), *n*	30,619	1352
Base model (y/BMI unit)	−0.17 (−0.18, −0.16)***	0.02 (−0.03, 0.06)
Adjusted model (y/BMI unit)	−0.17 (−0.18, −0.16)***	0.02 (−0.03, 0.06)
Replication cohort (born 1981–1996), *n*	1267	198
Base model (y/BMI unit)	−0.16 (−0.21, −0.11)***	−0.03 (−0.11, 0.05)
Adjusted model (y/BMI unit)	−0.17 (−0.21, −0.12)***	−0.03 (−0.11, 0.05)

^1^β-Values from linear regression analyses are given in years/BMI unit with 95% CIs in parentheses. The base model is unadjusted and the adjusted model is adjusted for birth year and country of birth.^***^P < 0.001. PHV, peak height velocity.

The identified threshold was close to the cutoff for overweight at 8 y of age both when using the cutoff from the International Obesity Task Force (18.44) (15) and when using the cutoff from the CDC (17.93) (16). Childhood BMI was inversely associated with age at PHV in normal-weight but not overweight boys (**Tables 3** and **4**). All observed associations were unaltered after adjustment for birth year and country of birth (Tables 2–4).

**TABLE 3:** Association between childhood BMI (in kg/m^2^) and age at PHV according to childhood weight status^1^

	Normal weight (BMI < 18.44)	Overweight (BMI ≥ 18.44)
Original cohort (born 1945–1961), *n*	30,647	1324
Base model (y/BMI unit)	−0.17 (−0.18, −0.16)***	0.02 (−0.02, 0.07)
Adjusted model (y/BMI unit)	−0.17 (−0.18, −0.16)***	0.02 (−0.02, 0.07)
Replication cohort (born 1981–1996), *n*	1268	197
Base model (y/BMI unit)	−0.16 (−0.21, −0.11)***	−0.02 (−0.10, 0.06)
Adjusted model (y/BMI unit)	−0.16 (−0.21, −0.11)***	−0.02 (−0.10, 0.06)

^1^β-Values from linear regression analyses are given in years/BMI unit with 95% CIs in parentheses. The base model is unadjusted and the adjusted model is adjusted for birth year and country of birth. Normal weight or overweight was categorized according to the cutoff at 8 y of age from the International Obesity Task Force (18.44) (15). ***P < 0.001. PHV, peak height velocity.

**TABLE 4:** Association between childhood BMI (in kg/m^2^) and age at PHV according to childhood weight status^1^

	Normal weight (BMI < 17.93)	Overweight (BMI ≥ 17.93)
Original cohort (born 1945–1961), *n*	29,966	2005
Base model (y/BMI unit)	−0.17 (−0.19, −0.16)***	0.01 (−0.03, 0.05)
Adjusted model (y/BMI unit)	−0.17 (−0.19, −0.16)***	0.01 (−0.03, 0.05)
Replication cohort (born 1981–1996), *n*	1215	250
Base model (y/BMI unit)	−0.16 (−0.22, −0.11)***	−0.02 (−0.10, 0.05)
Adjusted model (y/BMI unit)	−0.17 (−0.22, −0.12)***	−0.02 (−0.10, 0.05)

^1^β-Values from linear regression analyses are given in years/BMI unit with 95% CIs in parentheses. The base model is unadjusted and the adjusted model is adjusted for birth year and country of birth. Normal weight or overweight was categorized according to the cutoff at 8 y of age from the CDC (17.93) (16). ***P < 0.001. PHV, peak height velocity.

## DISCUSSION

In the present study we demonstrate that prepubertal BMI is inversely associated with age at pubertal timing in normal-weight but not overweight boys in 2 population-based cohorts of boys born before and during the obesity epidemic. In normal-weight boys, age at PHV is $\sim\!2$ mo earlier for every unit increase in prepubertal childhood BMI.

Using longitudinal height and weight data from 181 girls, Frisch and Revelle (17) showed that both early- and late-maturing girls reached menarche at the same mean weight but not the same mean height. After this finding they proposed the “critical body-weight hypothesis” stating that to initiate puberty, girls need to reach a critical level of body mass. This hypothesis was one of the first linking nutrition and body size to pubertal events. An association between a high prepubertal BMI or prepubertal obesity and early puberty in girls has been established in several studies (1, 3, 18). For boys, studies are scarce and the results inconsistent regarding the association between prepubertal childhood BMI and pubertal timing. In an American cross-sectional study comprising 439 boys, BMI was negatively associated with testicular volume and pubic hair development. Furthermore, testicular volume was smaller for obese boys compared with nonobese boys at the same skeletal age, suggesting delayed puberty for obese boys (19). A community-based, cross-sectional study of 3872 US boys reported that puberty was earlier in overweight boys whereas it was later in obese boys (20). Thus, conflicting findings have been reported from previous cross-sectional studies in boys. In the present well-powered longitudinal study, we establish that prepubertal BMI is inversely associated with pubertal timing in boys. Furthermore, through the use of nonlinear modelling, we demonstrate that prepubertal BMI associates with pubertal timing in normal-weight but not overweight boys.

Given the earlier pubertal timing in overweight compared with normal-weight girls, it has been assumed that fat mass drives the association between prepubertal BMI and pubertal timing in girls. A proposed mechanism was that the adipocyte-derived hormone leptin, reflective of the amount of fat mass, is involved (21). We, herein, present the novel observation that prepubertal BMI associates with age at pubertal timing in normal-weight but not overweight boys. We have also replicated our analyses with similar results in a younger cohort exposed to the obesogenic environment of the obesity epidemic over decades. One may speculate that a certain body mass (17) or BMI is required to initiate puberty and that an additional increase in BMI above this threshold does not affect pubertal timing further in boys. The underlying mechanism is unknown.

The limitations of this study are that we did not have detailed measurements of body composition of the children, and that we do not have data on girls. However, to our knowledge, the BEST cohort is the first large population-based cohort of males with information on childhood BMI and age at pubertal timing that captures the entire pubertal period. The strengths of the present study include the large population-based cohorts reflecting childhood BMI and pubertal timing before, as well as during, the obesity epidemic. Moreover, we used an objective assessment of pubertal timing in boys, and performed detailed statistical analyses including a nonlinear modelling of the association between BMI and age at pubertal timing.

In the present study we demonstrate that prepubertal BMI at 8 y of age is inversely associated with age at pubertal timing in normal-weight but not overweight boys through the use of 2 population-based cohorts born before and during the obesity epidemic. Further studies are warranted to elucidate the mechanisms behind the observed association.

## Notes

Supported by the Swedish Research Council and the Swedish Government (under the Agreement for Medical Education and Research), the Lundberg Foundation, the Torsten Söderberg Foundation, the Novo Nordisk Foundation, the Knut and Alice Wallenberg Foundation, and the Anna Ahrenberg Foundation.

MB and JMK contributed equally to this work.

Abbreviations used:

BEST: BMI Epidemiology Study
PHV: peak height velocity
PIN: personal identity number
